# Effect of Accident in Production of Gynecological Conditions

**Published:** 1914-01

**Authors:** James E. King

**Affiliations:** Buffalo, N. Y.; Attending Gynecologist Buffalo General and Erie County Hospitals


### BUFFALO MEDICAL JOURNAL

VOLUME 69 JANUARY, 1914 No. 6

### Effect of Accident in Production of Gynecological Conditions

JAMES E. KING, M. D., Buffalo, N. Y.
Attending Gynecologist Buffalo General and Erie County Hospitals.

THERE are probably no experiences more perplexing and trying to the conscientious surgeon than to be called upon, in certain cases of accidental injury, to determine the influence of the alleged injury as a cause of a physical condition or as a factor in producing subjective symptoms. Many such cases offer ground for an honest difference of opinion, and as a result a plaintiff with a just claim may fail to recover adequate damages, or as is more often the case, a corporation becomes the victim of a clever lawyer, willing medical experts and a prejudiced jury. Of all such cases there are probably none more difficult, and in which more injustice may be done plaintiff or defendant, than those in which the effect of injury upon the female pelvic organs come in question. It is in such instances that the medical expert has a wide latitude and the jury will often decide, not so much upon the merits of the case as upon the personality of the expert. It is not my purpose, however, to discuss the question of medical expert testimony, alluring as the subject is, but rather to consider some of the possible effects of accident and injury upon the female pelvic organs and the relation such injuries bear in suits to recover damages.

Usually the condition and symptoms referable to the pelvic organs are mentioned in the complaint incidentally in connection with other injuries, but occasionally the pelvic condition alone is made the basis for the damage claims.

For convenience in considering the subject, the effect of accident and injury may be classified as follows:

1. Disturbances of menstruation;
2. Production of displacements;
3. Production of abortion and premature labor;
4. Injuries by direct violence.

The conditions under the last heading will not be discussed, as they are rare, and when present are so obvious that there is seldom ground for dispute.

Disturbance of the menstrual function following accidents presents some interesting features. It is not uncommon and is often presented as evidence of injury. The character of menstrual disturbance may present many possible variations. It is seen both in the parous and the nulliparous. The character of the accident and the degree of injury bears no direct relation to the particular disturbance that may result. A shock or fright alone, without physical injury, is sufficient. On the other hand, many of these women will associate such symptoms with actual or supposed injury to the abdomen.

Among the common effects of accident upon menstruation may be mentioned an irregular bloody discharge which begins quite promptly after the injury. This may continue, with slight remissions, over a period of weeks or it may persist uninterrupted for a shorter time. Following this the menstruation will usually recur more frequently and be more profuse than usual. An illustrative case is that of a young healthy German girl, 24 years old, of normal menstrual habit. On attempting to board a trolley car, the car suddenly started and she was thrown to the pavement. She was removed to her boarding house and she began to flow almost immediately, although it was still ten days to her normal menstrual date. The bloody discharge continued with slight intermissions and varying in amount for several weeks. During the next six months the flow recurred at irregular intervals. It was nearly a year before the normal menstrual habit was re-established. A careful physical examination of this patient three days after the accident showed a normal pelvis. Aside from a few bruises there were no evidences of injury, but the young woman for many months presented in marked degree the various phases of traumatic neurasthenia. Occasionally a woman who is menstruating at the time of the accident will suddenly cease. By far the most common effect, however, is the simple disturbance of the normal menstrual date with increased flow. The period will anticipate the normal date from a few days to a week with more profuse and lengthened flow.

Pelvic examination of these women will often show no pelvic abnormality. Sometimes there will be tenderness of uterus and ovaries suggestive of general pelvic congestion. There may be pelvic symptoms that bear this out.

It is interesting to speculate as to the cause of such flowing following accident. With an existing fibroid or polyp an explanation would be at hand, but in cases of perfectly healthy women with normal menstrual habit we are obliged to look for the cause in some other influence. In turning to shock and fright for our explanation we find much to justify our regarding these factors as the underlying cause. That mental states may influence menstrual function we have abundant clinical evidence. The mental distress of the unmarried woman who fears she may be pregnant will not infrequently delay or suppress menstruation. Great and sudden grief may do the same thing. On the other hand menstruation may be brought on by grief, great joy or sustained and unusual mental effort.

It is difficult to explain altogether satisfactorily how mental states may thus influence menstrual function. It is probably through the sympathetics and vaso-motors controlling uterine circulation. Crile has shown by his work that shock and fright will produce marked changes in the brain cells and it is not unlikely that the disturbance of the vaso-motors has its basis in pathology. Certain it is that all of these patients show to varying degree that peculiar symptom-complex which is commonly termed traumatic neurasthenia and hysteria. Undoubtedly our greater knowledge will show these conditions to be an expression of brain pathology resulting from the fright and shock, and then it will be much easier to account for the various functional disturbances that are present in these cases. In the writer's opinion the disturbances of menstrual function following accident, in the absence of any pelvic lesion, should be classified with other evidences of the neurasthenic state.

Under our second heading, displacements of the uterus, we find more tangible evidence of injury. That such effects may result requires no argument. In the etiology of retroversion Hirst places falls and jolts second to child-bearing in frequency. In a given case of retroverted uterus it is often very difficult to determine what factors are remotely or directly responsible. A retroversion occurring as the result of a fall or jolt presupposes relaxed uterine supports. Normal average supports maintain the uterus in position under the most trying circumstances but with relaxed and stretched supports, displacement may occur as the result of a trivial cause. Retroversion as a result of accident may, therefore, be more reasonably expected to be seen in women who have borne children. There are certain women, however, who have never borne children but whose uterine supports seem to be congenitally weak. These women too will require but little to throw the uterus back. In any case where accident or fall is given as a cause of retroversion one should of course be reasonably certain that the uterus was in position previously. Every physician sees cases of symptomless retroversion, and even should pelvic symptoms develop after an accident and examination discover a retroverted uterus it would not necessarily follow that the accident caused the retroversion.

The writer has been concerned in a number of litigated cases where retroversion entered into the complaint. In only one case did there seem to be reliable evidence that the uterus was in position previous to the accident. This woman while walking to her seat in a Pullman coach was thrown violently to the floor by the impact of the locomotive in making its coupling. The family physician had confined this woman twice and had had occasion to examine her pelvis shortly before her accident. At this time the uterus was in proper position, as it always had been. Two weeks after the accident, during which time the patient was confined to her bed, the doctor examined her again and found that she had a third degree retroversion. Later at the time of my examination the uterus was still retroverted. The ability and honesty of purpose of the physician in this case can not be called into question, and no doubt the displacement occurred as the result of the fall. An instance illustrating how such a fall may cause displacement occurred in the writer's practice. A young married woman was being treated for some minor pelvic condition. As she lived only a short distance out of town she occasionally drove in for her treatment. On returning home upon one of these occasions the horse became frightened. The young woman jumped from the buggy and fell, striking on her back. Upon reaching home she was conscious of bearing down and backache. Four days later upon examination the uterus was found to be retroverted. This woman's uterus was in proper position on the day of her fall and there is no question but what the fall was wholly responsible.

The influence of retroversion as a factor with the jury in determining damages is probably very small. Obviously it should have no place in the case unless there be reliable evidence that the uterus was previously in normal position.

Prolapse is sometimes claimed to be the result of accident. A uterus whose points of attachment are weakened may and occasionally does come down as an acute prolapse following sudden straining effort. As a result of accident or injury such a prolapse must be very unusual. One woman known to the writer sued a corporation for injuries, among which, mentioned in the complaint, was a prolapse of the uterus. This woman was standing on her front steps when the defendants fired an overcharged blast opposite her house and she straightway fell off the steps. The complaint did not specify whether it was the blast *per se* or the fall that caused the prolapsus.

The effect of accident and injury upon pregnancy is frequently abortion and premature labor. There are three possible causes that may operate to produce it under these circumstances. The shock and fright acting through the sympathetics; the jars from falls and finally the effect of direct violence. In a given case it is not often easy to determine which one of these factors is responsible. The influence of the mind upon uterine contraction is frequently seen in the lying-in chamber. Fright and shock acting through the sympathetics may possibly stimulate uterine contractions, or in some manner not altogether clear it may even cause death of the foetus. The effect of jars and falls upon the pregnant uterus is much more easily understood. A slight separation of the decidua or placenta may be sufficient to induce contractions and expulsion of the uterine contents. Direct violence may act in the same way.

If miscarriage can be embodied in the complaint as due directly or indirectly to accident it usually means that the plaintiff will recover damages. A miscarriage appeals to the average jury mind. He knows what it means and he knows from hearsay and perhaps from family experience that shock and injury may produce it. The jury is therefore always ready to accept the expert's opinion upon that point. The question as to whether the miscarriage resulted from the accident is a point for the jury to decide and no matter how remote the miscarriage may be this point is usually decided in favor of the plaintiff.

Some of the suits in which miscarriage largely figures are interesting. They illustrate the views of the average jury upon the subject. A pregnant woman and her brother were driving over a bridge. The team fell partially through a defective spot. The occupants of the buggy were not thrown out or injured in any way, but the woman was much frightened. She rendered what little aid she could and then ran a considerable distance for assistance. She miscarried some time later and recovered damages from the town.

Another suit based upon somewhat similar claim was that of a pregnant woman who recovered from a street railway company for a miscarriage caused by fright. The plaintiff stood upon or near the track waiting for a car when a car, beyond control, dashed down the hill and the woman had only time to jump from its path. Although not struck she fainted from fright. She miscarried and recovered from the company. Still another successful suit was that of a woman in a railway collision. There was no claim made for injuries but later she gave birth to a premature still-born infant. The claim that the miscarriage was the result of the terror and alarm at the time of the accident was shared by the jury and the plaintiff recovered from the railroad. One other example is interesting. A pregnant woman driving over a low bridge was overturned into the ditch below because of some defect. A few weeks later a premature labor resulted in the birth of a pair of twins which lived but a few hours. The plaintiff sued and won and was compensated for the *pain and distress* she suffered as a result of the premature labor.

The cases quoted are taken from court records and clearly indicate how willing a jury is to compensate a woman for a miscarriage, no matter how remotely it may have been due to the accident. It is an interesting and curious fact that apparently the offspring has no value in the eyes of the law, and yet one of the most serious crimes on the calendar is the destruction of the child in utero. The woman may be compensated for the pain and suffering incident to a miscarriage but not for the loss of her offspring.

